# Liver fibrosis and MAFLD: the exploration of multi-drug combination therapy strategies

**DOI:** 10.3389/fmed.2023.1120621

**Published:** 2023-04-20

**Authors:** Qingfu Dong, Haolin Bao, Jiangang Wang, Wujiang Shi, Xinlei Zou, Jialin Sheng, Jianjun Gao, Canghai Guan, Haoming Xia, Jinglin Li, Pengcheng Kang, Yi Xu, Yunfu Cui, Xiangyu Zhong

**Affiliations:** ^1^Department of Hepatopancreatobiliary Surgery, The Second Affiliated Hospital of Harbin Medical University, Harbin, Heilongjiang, China; ^2^Department of General Surgery, Tangdu Hospital, Air Force Medical University, Xi'an, Shaanxi, China; ^3^Key Laboratory of Basic Pharmacology of Ministry of Education, Zunyi Medical University, Zunyi, Guizhou, China; ^4^Key Laboratory of Functional and Clinical Translational Medicine, Fujian Province University, Xiamen Medical College, Xiamen, Fujian, China; ^5^Jiangsu Province Engineering Research Center of Tumor Targeted Nano Diagnostic and Therapeutic Materials, Yancheng Teachers University, Yancheng, Jiangsu, China; ^6^Key Laboratory of Biomarkers and In Vitro Diagnosis Translation of Zhejiang Province, Hangzhou, Zhejiang, China; ^7^Key Laboratory of Gastrointestinal Cancer, Ministry of Education, School of Basic Medical Sciences, Fujian Medical University, Fuzhou, Fujian, China; ^8^State Key Laboratory of Chemical Oncogenomics, Key Laboratory of Chemical Genomics, Peking University Shenzhen Graduate School, Shenzhen, Guangdong, China; ^9^Department of Pathology, Li Ka Shing Faculty of Medicine, The University of Hong Kong, Hong Kong, China; ^10^Key Laboratory of Intelligent Pharmacy and Individualized Therapy of Huzhou, Department of Pharmacy, Changxing People's Hospital, Changxing, Zhejiang, China

**Keywords:** liver fibrosis, metabolic-associated fatty liver disease (MAFLD), non-alcoholic steatohepatitis (NASH), cirrhosis, drug combination therapy

## Abstract

In recent years, the prevalence of metabolic-associated fatty liver disease (MAFLD) has reached pandemic proportions as a leading cause of liver fibrosis worldwide. However, the stage of liver fibrosis is associated with an increased risk of severe liver-related and cardiovascular events and is the strongest predictor of mortality in MAFLD patients. More and more people believe that MAFLD is a multifactorial disease with multiple pathways are involved in promoting the progression of liver fibrosis. Numerous drug targets and drugs have been explored for various anti-fibrosis pathways. The treatment of single medicines is brutal to obtain satisfactory results, so the strategies of multi-drug combination therapies have attracted increasing attention. In this review, we discuss the mechanism of MAFLD-related liver fibrosis and its regression, summarize the current intervention and treatment methods for this disease, and focus on the analysis of drug combination strategies for MAFLD and its subsequent liver fibrosis in recent years to explore safer and more effective multi-drug combination therapy strategies.

## Introduction

With the prevalence and development of the disease, non-alcoholic fatty liver disease (NAFLD) has become the most general etiology of chronic liver disease worldwide. As one of the most common indications for liver transplantation (1, 2), it affects about 25% of the world's population, and its prevalence continues to increase (3). Increasing number of studies have reported that metabolic dysfunction, including obesity, type 2 diabetes mellitus (T2DM), hypertension, and metabolic syndrome, is closely associated with the complex pathological mechanism of NAFLD (4). To better integrate the present understanding of the heterogeneity of NAFLD patients, reflect the pathogenesis more accurately, realize stratified management of patients, and accelerate the translation of new treatments, in 2020, an expert group proposed the new nomenclature “metabolic-associated fatty liver disease (MAFLD)” (5), which is now globally multi-stakeholder-agreed (6). From metabolic overload to durative hepatocyte injury, MAFLD will eventually lead to liver fibrosis, cirrhosis, and even HCC. In addition, the illness is closely related to various extrahepatic diseases, such as chronic kidney disease and cardiovascular complications (7, 8). Multiple factors, including race, age, sex, hormonal status, metabolic rate, diet, alcohol consumption, cigarette smoking, genetic predisposition, and microbiota, may influence the heterogeneity of disease progression and clinical manifestations in MAFLD (5). Consequently, efficacious treatments must consider various complex factors and may require personalized multi-drug combination therapies. This review introduces the mechanism of MAFLD-associated liver fibrosis progression and regression, discusses the role of lifestyle intervention, bariatric metabolic surgery, liver transplantation, and drug therapy, and focuses on analyzing drug combination therapy related to MAFLD and liver fibrosis in recent years. It aims to explore more effective multi-drug combination strategies for treating MAFLD and its related liver fibrosis and reducing the disease burden.

## MAFLD-related liver fibrosis and its regression mechanism

**Figure 1:**
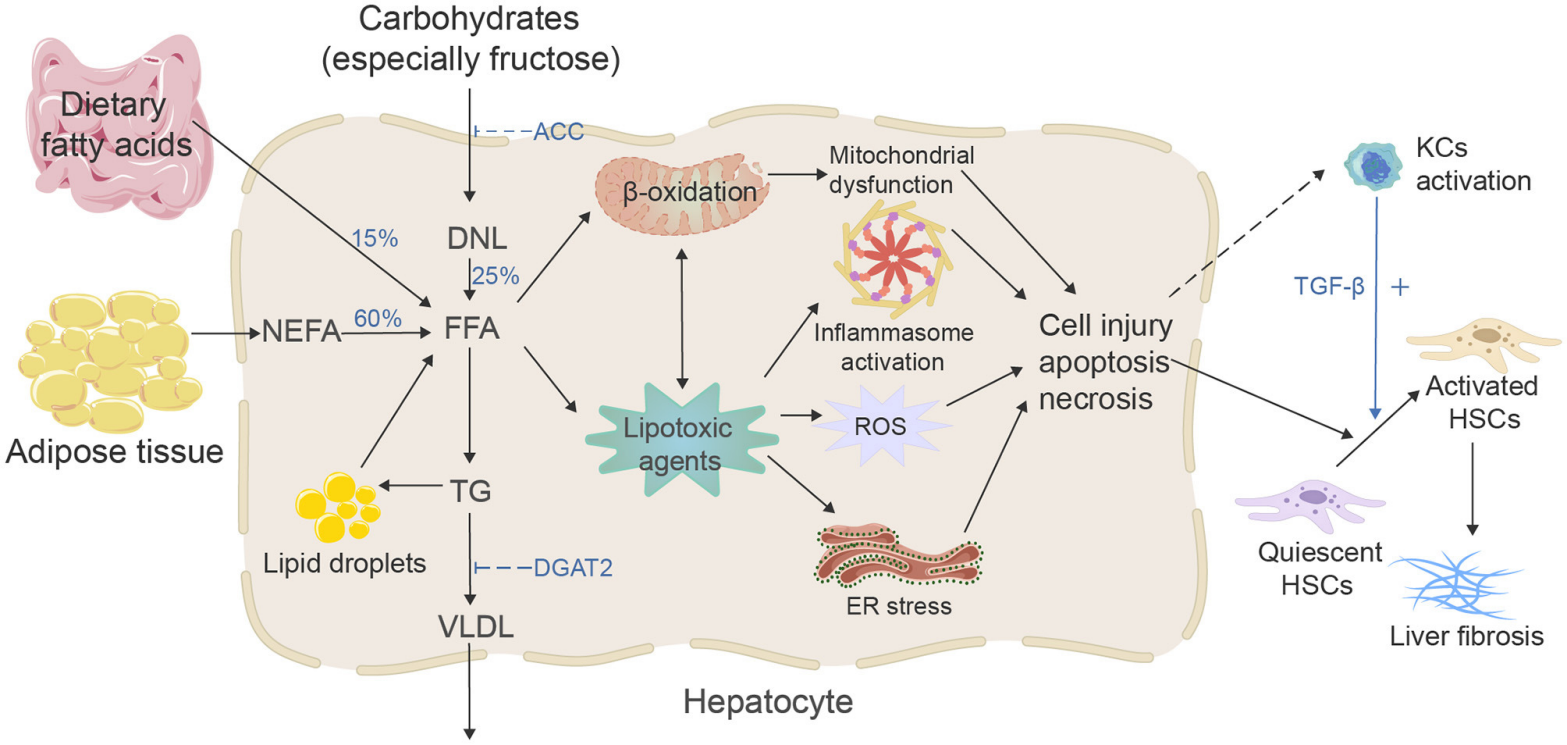
Mechanisms of MAFLD-related liver fibrosis. Free fatty acids (FFAs) and their metabolites may represent the lipotoxic agents that induced the development of MAFLD, and the increasing concentration of FFA is considered the most critical stage. There are three primary sources and two fates of hepatic FFA. Excessive FFA and their metabolites will cause mitochondrial dysfunction, stimulate ER stress, oxidative stress, and inflammasome activation, then activate the HSCs and eventually lead to liver fibrosis.

During the past two decades, the incidence of HBV and HCV-related liver fibrosis and liver cancer has declined due to vaccination and new effective antiviral treatments. However, as the prevalence of MAFLD has reached pandemic levels, the incidence of MAFLD-related liver fibrosis is increasing (9–11). At the same time, studies have shown that the progression of liver fibrosis is significantly related to an increased chance of hepatocellular carcinoma (HCC); MAFLD has risen as one of the major causes of HCC (12); in addition, the increased risk of severe liver-related and cardiovascular events in MAFLD patients is closely related to the fibrotic stage (13) and is the strongest predictor of mortality in MAFLD patients (14), so it is crucial to understand the mechanism of MAFLD-related liver fibrosis (Figure 1) and explore effective antifibrotic therapeutic strategies. From metabolic disorders of fatty acids and carbohydrates to persistent liver injury, ultimately leading to liver fibrosis and cirrhosis, the pathogenesis of MAFLD-associated fibrosis relates to many complicated drivers and diverse mechanisms, such as high-concentration hepatic free fatty acid (FFA)-induced mitochondrial dysfunction, oxidative stress, endoplasmic reticulum (ER) stress and inflammation, subsequent hepatocyte apoptosis, and extracellular matrix (ECM) formation, which also involves the interaction of immunity and genetic and epigenetic regulations (15, 16). To date, the increase in hepatic FFA concentration is still considered the most critical stage in the development of MAFLD and activating of hepatic stellate cells (HSCs) is the key pathogenic event for the development of liver fibrosis (17). FFA comes from three sources (18): 15% comes from dietary fat absorbed in the gut, and bile acids play a crucial role in lipid absorption (19); 25% comes from *de novo* lipogenesis (DNL) of new fat synthesis, in which liver cells generate new fatty acids by converting excess carbohydrates (especially fructose), and acetyl-CoA carboxylase (ACC) is a crucial enzyme in the regulation of DNL, catalyzing the acetyl-CoA converse into malonyl-CoA; and 60% of fatty acids come from the non-esterified fatty acid pool or lipolysis of triglyceride (TG) in adipose tissue. In the hepatocytes, fatty acids' two significant fates are mitochondrial β-oxidation and re-esterification to form TG. A part of TG can be converted to very low-density lipoprotein (VLDL) and transported into the blood. Another part of TG is stored in lipid droplets, which undergo regulated lipolysis and release fatty acids into FFA pools (15). It has been suggested that FFA and its metabolites may represent the lipotoxic agents responsible for the development of MAFLD (20). When fatty acids are redundant, or their processing is impaired, they may serve as substrates to generate lipotoxic lipids that stimulate the ER stress, oxidative stress, and inflammasome activation, and release danger-associated molecular patterns, which lead to liver cell damage and induce diverse modes of cell death, including apoptosis and necrosis (15). Damaged hepatocytes can activate HSCs via paracrine signals. For example, lipotoxic hepatocytes can mediate the activation and proliferation of HSCs by producing exosomes, such as exosomal miR-27a and exosomal miR-1297 (21, 22). IL11 from lipotoxic hepatocytes stimulates HSCs to myofibroblast transformation in a paracrine manner (23). Lipotoxic-related reactive oxygen species (ROS) production in hepatocytes is a critical factor in the activation of HSCs in fibrosis (24, 25). The mitochondrial dysfunction, production of ROS, ER stress, and sterile hepatocyte death conduce to the pro-inflammatory environment of the liver, contributing to the pro-inflammatory environment. Bacterial translocation due to intestinal barrier dysfunction can induce an inflammatory response (26). Neutrophils remove apoptotic liver cells and produce various cytokines to participate in the occurrence of liver fibrosis. The transforming growth factor-β (TGF-β) from Kupffer cells is the most influential profibrotic factor (27). It should be noted that during MAFLD, immune mechanisms link the metabolic injury to inflammation and fibrosis; susceptibility to inflammatory liver states is also closely related to genetic and epigenetic backgrounds (16, 28, 29). During the injury–repair response, activated HSCs migrate to the injury site. They secrete ECM, accumulating and eventually forming fibrous scars and regenerative nodules that replace the damaged normal tissue, resulting in portal hypertension and cirrhosis. From an asymptomatic to a symptomatic phase (decompensated cirrhosis), associated complications often lead to hospitalization, poor quality of life, and higher mortality (30).

Metabolic-associated fatty liver disease-related liver fibrosis regression has been verified in many animal experiments and clinical practice (31), and serial liver biopsy has proved that bariatric metabolic surgery can effectively promote the regression of liver fibrosis in patients with non-alcoholic steatohepatitis (NASH) (32). After the etiology of chronic injury is eliminated, liver fibrosis stops progressing or even regresses, which is related to many mechanisms. Eliminating the etiology of chronic liver injury is the vital goal of antifibrotic therapy. However, not all causes of chronic liver injury can be effectively removed, especially for MAFLD-related liver fibrosis. In addition, direct anti-fibrosis or reverse fibrosis therapeutics are more hopeful strategies for patients with severe liver cirrhosis. Therefore, we need to deeply understand the mechanism of liver fibrosis regression to explore effective therapeutic targets. The mechanisms of liver fibrosis reversibility include HSCs' inactivation and apoptosis and the fibrous scar's resorption (33). First, reducing activated HSCs is an essential target of antifibrotic therapy. On the one hand, we can reduce the number of HSCs by promoting senescence and apoptosis. Antiretroviral drugs against HIV can enhance the proliferation of hepatocytes and the apoptosis of HSCs (34). In addition, studies have shown that TNF-α activates a nuclear factor-κB-dependent gene program to promote HSCs survival and differentiation (35), which provides a possible target for us to explore the senescence and apoptosis of HSCs and inhibit the NF-kB pathway that may inhibit liver fibrosis by inducing apoptosis of HSCs. On the other hand, HSCs activation can be inhibited or reversed to fight liver fibrosis. Some studies have shown that peroxisome proliferator-activated receptor gamma (PPARγ) is a crucial mediator of HSCs activation and phenotypic changes and can affect the state of HSCs in the quiescent phase. The addition of PPARγ agonists *in vitro* and *in vivo* can reduce the activation of HSCs and promote the degradation of the ECM (36). Moreover, reducing collagen production, enhancing ECM degradation, and changing ECM's spatial conformation and matrix stiffness are also exploration targets for antifibrotic therapy (37, 38). For example, a study has shown that lysyl oxidase-like 2 (LOXL2) monoclonal antibodies can alleviate liver fibrosis and promote fibrosis reversal in mice (39).

## Interventions/treatment measures for MAFLD and its related liver fibrosis

### Lifestyle intervention

The development of MAFLD is closely related to lifestyle factors, especially excess caloric intake paired with insufficient physical exercise (40). Current studies suggest that dietary intervention improves MAFLD with or without physical activity, and training also reduces hepatic steatosis with or without dietary intervention (41). The 2018 ASSLD Practice Guidelines state that a combination of a low-calorie diet and moderate-intensity exercise may lead to sustained weight loss over time, with a 3–5% weight loss improving steatosis and a 7–10% weight loss improving fibrosis (42). However, relevant clinical trials found that most patients could not achieve the level of weight loss that can improve liver fibrosis (43). It is difficult to achieve the goal of enhancing fibrosis through lifestyle intervention alone. A recent prospective cohort study found that healthy lifestyles positively correlate with all-cause mortality in MAFLD patients (44). The COVID-19 pandemic has affected people's lifestyles seriously, and new MAFLD diagnoses have increased during the pandemic. A retrospective study including 973 participants found that before the pandemic (2018–2019), the independent lifestyle predictor of MAFLD was regular late-night eating, while in the epidemic (2019–2020), it was higher daily alcohol intake (45). The research in mice showed that respiratory exposure to silica nanoparticles induces hepatotoxicity, resulting in inflammatory infiltration, and even causes the deposition of collagen (46). Another cross-sectional study conducted in China proves that the rising sickness rate of MAFLD in the real world is significantly related to long-term exposure to ambient PM1, PM2.5, PM10, and NO2, particularly those who are men, alcohol drinkers, cigarettes smokers, high-fat diet consumers, and central obesity (47). Lifestyle changes make MAFLD more and more common. There is no doubt that healthy lifestyles can help prevent the occurrence of MAFLD, and timely change in unhealthy lifestyles is very important for MAFLD patients. However, as a preventive strategy that can be extended to the whole population, lifestyle interventions alone have yet to control the prevalence of MAFLD and the development of MAFD-related liver fibrosis, so we must actively explore other preventive and therapeutic measures.

### Bariatric metabolic surgery

Bariatric metabolic surgery is now recommended as an effectual treatment for clinically severe obesity and its interrelated comorbidities and has generally been accepted by patients in recent years (48). In a bariatric metabolic surgery center in France, Guillaume Lassailly conducted a long-term follow-up on 180 severely obese patients who were biopsy-confirmed with NASH and underwent bariatric metabolic surgery; they found that 84% of the participants had regression of NASH in liver samples after 5 years, indicating that the fibrosis of the liver was reduced from the first to the fifth year (32). Although it is generally believed that MAFLD is closely related to obesity, there is growing evidence proving that not all overweight individuals have MAFLD. Approximately 40% of MAFLD patients are classified as non-obese (49, 50). Does bariatric metabolic surgery have a therapeutic effect on low BMI MAFLD patients? Adrian T Billeter researched the curative effect of Roux-en-Y gastric bypass (RYGB) in advanced MAFLD; 20 patients participated in this prospective trial and underwent RYGB surgery; liver biopsy was performed during the operation and followed up 3 years later. The results showed that after 3 years of RYGB treatment, MAFLD completely disappeared in all patients, and fibrosis was also improved, 55% of the patients stopped insulin therapy, glycosylated hemoglobin decreased significantly, new lipogenesis decreased, β-oxidation was enhanced, and finally, the secretion of gastrointestinal hormones and adipokines was favorably altered (51). The aforementioned results suggest that bariatric metabolic surgery positively affects MAFLD regardless of obesity, but there are still certain risks in these surgical treatments for MAFLD. For example, the mortality rate of patients with decompensated liver cirrhosis after bariatric metabolic surgery is as high as 16.3% (52). Therefore, bariatric metabolic surgery is excluded as the first-line treatment of MAFLD. It is believed that with the continuous improvement of relevant clinical research, clinicians can more accurately evaluate the pros and cons of bariatric metabolic surgery for MAFLD, better grasp the indications for surgical treatment, and make more patients benefit from it.

### Liver transplantation

Because of the severe scarcity of liver resources, the high cost of liver transplantation (LT), and a series of problems, such as immune rejection after transplantation, LT is just considered for advanced MAFLD patients with severe complications in most cases. However, it cannot be ignored that MAFLD is the fastest-rising indication for LT in Western countries. Its interrelated end-stage liver disease and HCC have grown to be LT's common indications worldwide (2). Severe cirrhosis, liver failure, and severe portal hypertension caused by advanced liver fibrosis usually require LT. Some patients with non-resettable HCC also need LT for better treatment (53, 54). Although the survival rate of liver transplant recipients with MAFLD is similar to that of liver transplant recipients with other etiologies, liver transplant recipients still seem prone to relapse MAFLD due to the persistence of diseases such as metabolic syndrome (55). This proportion is as high as 78–88%, usually relapsing within the first 5 years after LT. However, ~11–14% may develop cirrhosis within 5 years after LT (56). As the number of liver transplant recipients continues to increase, their quality of life continues to improve, their survival time continues to increase, and increased attention has been paid to the occurrence of MAFLD after transplantation. In addition, due to the shortage of liver resources and the prevalence of MAFLD, some donor livers with steatosis also need to be used in LT.

### Pharmacological treatment

Pharmacological treatment is very attractive to MAFLD and liver fibrosis, not only due to its convenience but also because various mechanisms of disease progression can be targeted. At present, many drugs are actively developed for the therapy of MAFLD and its related liver fibrosis, which are mainly divided into the following categories according to the main mechanism (18, 57): The first category is agents acting on lipid syntheses and fat accumulation, such as glucagon-like peptide 1 (GLP-1) agonists, ACC inhibitors, Fanitol X receptor (FXR) agonists, and PPAR-α/δ agonists. The second category is drugs that act on cellular stress and apoptosis, including vitamin E and caspase inhibitors. The third category is drugs that play roles in the immune and inflammatory response, such as C-C chemokine receptor type 2 and type 5 antagonists. The fourth category is drugs that directly target the fiber formation process, such as LOXL2 monoclonal antibodies. In addition, new studies have also found that anti-angiogenic drugs can improve liver fibrosis, such as recombinant vascular endothelial growth factor (rVEGF) and bevacizumab (58). These drugs have some efficacy in the therapy of MAFLD and its associated liver fibrosis. However, few pharmacological treatments reached satisfactory endpoints assessed by liver biopsy or with negligible side effects in clinical trials (18). It is essential to accelerate the discovery of new pharmacotherapeutics and explore better multi-drug combination therapies.

In summary (Table 1), measures such as lifestyle intervention have failed to effectively control the increasing prevalence of MAFLD and the progression of liver fibrosis. Bariatric metabolic surgery is still not suitable as the first-line treatment for MAFLD. LT, an option method to save lives for patients with MAFLD-related non-resettable HCC or end-stage liver diseases, is not a good solution for decreasing the burden of MAFLD and its associated liver fibrosis. In addition, the advance of MAFLD-related liver fibrosis involves numerous complicating factors, and the impact of single-drug therapy is very limited. Effective pharmaceutical therapies may need to consider multiple mechanisms, such as metabolic disorders, inflammation, immunity, and fibrosis. Combination pharmaceutical therapies may be an inevitable choice to achieve adequate control of MAFLD and its related liver fibrosis in the future.

**Table 1:** Comparison of different therapeutics for MAFLD.

**Therapeutics**	**Superiorities**	**Shortcomings**
Lifestyle intervention	Applicable to the whole population Reduces all-cause mortality	Difficult to persist Failed to control the prevalence of MAFLD effectively
Bariatric metabolic surgery	Surgery is becoming less invasive Gets rid of taking medicines every day	Risk of post-operative complications Lack of adequate clinical research Is excluded as the first-line treatment of MAFLD
Liver transplantation	A life-saving method	Expensive Lack of liver resources Immune rejection, MAFLD relapsing
Pharmacological therapies	Convenient Affordable Various mechanisms of disease progression can be targeted	No specific drugs Drug side effects The impact of single-drug therapy is minimal

## Multi-drug combination therapies

In the following, we will analyze some pharmaceutical combination therapies for MAFLD and liver fibrosis in recent years (Table 2).

**Table 2:** Some combination treatments for MAFLD and liver fibrosis in recent years.

**Agents**	**Primary mechanism**	**Patients**	**Pros and cons of combined therapy**	**NCT number (and Phase)**
Cilofexor + firsocostat	FXR agonist ACC inhibitor	392 patients with bridging fibrosis or compensated cirrhosis (F3–F4)	Combined therapy has better anti-fibrosis potential but still induces hypertriglyceridemia	NCT03449446 (Phase 2b)
Cilofexor + firsocostat + semaglutide	FXR agonist ACC inhibitor GLP-1 receptor agonist	Patients with NASH	Cilofexor and firsocostat-induced hypertriglyceridemia is alleviated by semaglutide	NCT03987074 (phase 2)
Cilofexor + firsocostat + fenofibrate	FXR agonist ACC inhibitor PPARα agonist	Patients with NASH with elevated TG (≥150 and < 500 mg/dL)	Fenofibrate was safe and effectively mitigated increases in TG associated with ACC inhibitor	NCT02781584
PF-05221304 + PF-06865571	ACC inhibitor DGAT2 inhibitor	Adults with NAFLD	ACC inhibitor-mediated serum TG elevation was mitigated	NCT03776175 (phase 2a)
OCA + atorvastatin	FXR agonist HMGR inhibitors	84 participants with NASH	Atorvastatin attenuates OCA-induced LDL-C elevation	NCT02633956 (Phase 2)
Pioglitazone +tofogliflozin	PPARγ agonist SGLT-2 inhibitor	Patients with NAFLD with T2DM and a hepatic fat fraction of ≥10%	Therapeutic potential to prevent the progression of NASH to HCC	/
HXT + vitamin E	Natural compounds Antioxidant	Children with biopsy-proven NAFLD	Ameliorate steatosis and hypertriglyceridemia, reducing the fibrosis stage	NCT02842567

### Combination therapy based on glucagon-like peptide 1 receptor agonists

Glucagon-like peptide 1 (GLP-1), a pleiotropic peptide hormone secreted by intestinal L cells (59), controls insulin hormone secretion, intestinal motility, and body weight. GLP-1 receptor agonists, developed for treating T2DM and obesity recently, have demonstrated a favorable benefit and decreased the occurrence of cardiovascular-related adverse events in T2DM patients. The current analysis considers all people with T2DM, and people with a liver fat content of >5% are deemed to have MAFLD (60). MAFLD increases cardiovascular morbidity and mortality (18). In recent years, the potential role of the combination therapy of GLP-1 receptor agonists and sodium–glucose cotransporter-2 (SGLT-2) inhibitors in treating MAFLD has attracted increased attention. Numerous clinical trials link this combination treatment to reductions in intrahepatic triglyceride accumulation and liver fibrosis, even though none of the GLP-1 or SGLT-2 receptors are expressed in the liver (61). Therefore, GLP-1 receptor agonists are a potentially valuable element of combination therapy to address different complementary pathways in MAFLD therapy (62). For example, in a 24-week exploratory phase 2 trial, the GLP-1 receptor agonist semaglutide alleviates cilofexor and firsocostat-induced hypertriglyceridemia, resulting in more significant reductions in liver enzymes, liver fat, and non-invasive imaging assessed liver fibrosis (NCT03987074) (63). In conclusion, GLP-1 receptor agonists are very suitable as the primary drugs for the combination therapy of MAFLD characterized by metabolic disorders.

### Combined use of acetyl-CoA carboxylase inhibitors

Acetyl-CoA carboxylase (ACC) is a critical enzyme in *de novo* lipogenesis (DNL), catalyzing the rate-limiting step in converting acetyl-CoA to malonyl-CoA, regulating the fatty acids' mitochondrial β-oxidation and playing a vital role in the accumulation of TG in hepatocytes. Animal studies have confirmed that inhibiting ACC in rat models can reduce liver fibrosis (18). Currently used ACC inhibitors mainly include firsocostat (formerly GS-0976) and PF-05221304. These two acetyl-CoA carboxylase inhibitors affect serum TG. In a study of NASH patients, it was found that treating with GS-0976 20 mg per day for 12 weeks reduced liver steatosis, selective markers of liver fibrosis, and biochemistry but caused significant increases in serum TG levels in most patients (64); this asymptomatic hypertriglyceridemia can be partially resolved by fibrate (belonging to PPARα agonists). Lawitz EJ compared the curative effects of Vascepa or fenofibrate in mitigating triglyceride elevation in patients with NASH treated with cilofexor and firsocostat. NASH patients with elevated TG were randomly divided into two groups: one group treated with Vascepa 2 g twice a day for 2 weeks, and another with fenofibrate 145 mg once a day; both groups followed these with cilofexor 30 mg and firsocostat 20 mg once a day for 6 weeks, then safety, blood lipids, and liver biochemistry were monitored. After 6 weeks of combination therapy, fenofibrate has a better curative effect than Vascepa in reducing elevated TG in patients (NCT02781584) (65). Similarly, the ACC inhibitor PF-05221304 alone significantly reduced hepatic steatosis and induced an asymptomatic increase in serum TG levels; the latter may represent an adverse cardiometabolic profile limiting the long-term use of this class of drugs (66). Diacylglycerol acyltransferase 2 (DGAT2) is an enzyme that catalyzes the last step of TG synthesis. It plays a role in regulating VLDL production in rodents (67); PF-06865571 is an inhibitor of DGAT2 (68). In a Phase 2a pilot study combining PF-05221304 and PF-06865571, a significant attenuation of ACC inhibitor-mediated effects on serum TG was observed (NCT03776175) (69). We look forward to longer-period research including liver biopsies to further demonstrate the impact of co-administration of PF-05221304 and PF-06865571 on NASH regression and fibrosis in NASH patients. In summary, ACC inhibitors are currently attractive target drugs to restore the balance of hepatic fatty acid metabolism in patients with MAFLD. Combined use with drugs, such as FXR agonists, can reduce liver fibrosis, but attention still needs to be paid to the combination use of other medications that regulate lipogenesis to minimize the impact on blood lipids.

### Combination of Fanitol X receptor agonists and other drugs

Fanitol X receptor (FXR) is a nuclear receptor abundantly expressed in the liver and intestinal epithelia, which is vital in the perception of bile acid signals. It regulates inflammatory pathways by reducing pro-inflammatory cytokines, inhibiting the activation of inflammasomes, and upregulating anti-inflammatory mediators (70). Studies have confirmed that activating the FXR in HSCs can reduce the HSCs' response to profibrotic signals such as TGFβ, thereby decreasing ECM formation and inhibiting the development of fibrosis (71). FXR agonists mainly include obeticholic acid (OCA) and cilofexor. One serious limitation of the OCA therapy is dyslipidemia (elevated low-density lipoprotein cholesterol), which may lead to a rising risk in NASH patients with atherosclerosis. The 3-hydroxy-3-methylglutaryl-CoA reductase (HMGR) plays a pivotal role in the biosynthesis of cholesterol; HMGR inhibitors inhibit cholesterol synthesis by atorvastatin (72). Clinical research about the combination of OCA and a statin found that atorvastatin attenuated OCA-induced low-density lipoprotein cholesterol (LDL-C) elevation in patients with NASH after 16 weeks of treatment (NCT02633956) (73). Cilofexor can significantly reduce liver steatosis, biochemical markers, and serum bile acid levels. Studies have shown that it has better anti-fibrosis profit when combined with ACC inhibitor firsocostat but still faces the problem of hypertriglyceridemia (NCT03449446) (53). This issue limits the application of this pharmacological combination therapy strategy. Therefore, it is necessary to explore more precise and effective drug targets; otherwise, multi-drug combination therapies are needed. As previously mentioned, adding fenofibrate or semaglutide relieves elevated TG induced by the combination therapy of cilofexor and firsocostat (63, 65). In addition, the FXR receptor agonist tropifexor and the C-C chemokine receptor type 2 and type 5 antagonist cenicriviroc target steatosis, inflammation, and fibrosis pathways involved in MAFLD. FXR agonists, which restore bile acid metabolism and suppress inflammation, are essential to future combination therapy for MAFLD.

### Peroxisome proliferator-activated receptor modulators in combination with other drugs

Peroxisome proliferator-activated receptors (PPARs) are a class of nuclear hormone superfamily receptors widely involved in regulating inflammatory responses and metabolic homeostasis. Some agonists targeting PPAR combined with other different classes of drugs have a complementary effect in treating liver fibrosis, such as fenofibrate mentioned above (65, 74). In addition, pemafibrate is a selective PPARα modulator, and clinically relevant doses of pemafibrate were demonstrated to effectively and safely lower serum TG in mice (75). A study containing 118 patients evaluated the therapeutical effect of pemafibrate for MAFLD patients and showed that pemafibrate reduced liver stiffness but had no effect in reducing liver fat content (76). Pemafibrate may be a hopeful drug for treating MAFLD combined with medicines to lower hepatic fat. Another trial, including 70 participants with ultrasound-confirmed MAFLD, showed that the combination of ezetimibe plus rosuvastatin lowered hepatic fat (77). Further studies are needed to determine whether the combined use of pemafibrate, ezetimibe, and rosuvastatin can achieve more clinical benefits. In addition, a mouse model study showed that, at the two-time points of onset of NASH progression and HCC survival, combined treatment with pemafibrate and tofogliflozin (an SGLT-2 inhibitor) not only significantly relieved hyperglycemia and hypertriglyceridemia but also reduced ballooning of hepatocytes, reduced expression of ER stress-related genes level (such as Ire1a, Grp78, Xbp1, and Phlda3), and significantly improved the survival rate and decreased the tumors' numbers in the liver. It suggests that PPARα modulator and SGLT-2 inhibitor combined treatment has the potential to inhibit the progression of NASH to HCC (78). Pioglitazone belongs to the first-generation thiazolidinediones, which is a PPARγ agonist, and it was proved that pioglitazone could improve liver fibrosis scores in non-diabetic patients with NASH (79). In patients with T2DM and MAFLD, 32 suitable patients were treated with pioglitazone and tofogliflozin; compared with every single-drug therapy group, combination therapy gained additional improvement in HbA1C. Weight gain mediated by pioglitazone was reduced with the concomitant use of tofogliflozin (80). In conclusion, agonists of PPAR are widely involved in regulating metabolic homeostasis and inflammatory response, have therapeutic potential in preventing the progression of MAFLD to HCC, and have a prominent position in combination therapy.

### Combination therapy of natural compounds

Up to now, there is no specific clinically useful therapy for MAFLD, thus some people try to screen and study natural products or synthetic compounds to find efficacious drugs for the treatment of MAFLD, such as the natural sesquiterpene ketone (Nok) (81), hydroxytyrosol (HXT), and vitamin E. Both HXT and vitamin E have good antioxidant properties (82), and oxidative stress is an influential factor that induces HSCs to activate and leads to liver fibrosis (83). HSCs can be activated by tumor growth factor TGF-β, leading to a significant increase in proliferation rate (84). Nadia Panera used this cell as an *in vitro* model to conduct experiments, indicating that the use of HXT and vitamin E alone or in combination treatment resulted in a marked decrease in this TGF-β-dependent pro-proliferative effect. The combined therapy of HXT + vitamin E more effectively inhibited the impact of TGF-β on HSCs. HXT + vitamin E significantly reduced the pattern of liver fibrosis observed in a mouse model which was fed a carbon tetrachloride plus Western diet (82). In addition, in children with biopsy-proven MAFLD, a 4-month-old short-term HXT + vitamin E treatment responds to DNA damage recovery by increasing circulating IL-10 levels, ultimately ameliorating steatosis and hypertriglyceridemia, reducing the fibrosis stage in children with MAFLD, and this beneficial effect is extended over time (NCT02842567) (85). Screening and research on natural products or synthetic compounds to treat MAFLD will help explore new antifibrotic therapeutic targets, which may provide new elements for pharmaceutical combination therapies.

### Combined use of different endothelial cell modulators

Liver fibrosis is due to the excessive formation of extracellular matrix, often accompanied by neovascularization and changes in vascular structure, ultimately causing organ injury and failure (86, 87). In recent years, angiogenesis inhibitors such as bevacizumab have made great progress in the treatment of tumors (88). Simultaneously, people are also actively exploring the application of angiogenesis modulators in treating liver fibrosis. The microvessels in the liver contain portal veins, hepatic sinusoids, and central vessels, and different vessels play different roles in the development of liver fibrosis (58). Therefore, achieving effective anti-fibrosis through targeted vascular therapy may require a combination of varying angiogenesis modulators. Leukocyte cell-derived chemotaxin 2 (LECT2), a newly discovered hepatic factor, is significantly increased in MAFLD patients (89). Ec-specific receptor Tie1 is necessary for the maturation of blood vessels. Meng Xu found that direct binding of LECT2 to Tie1 can inhibit portal vein angiogenesis, induce hepatic sinusoidal capillarization, and promote liver fibrosis. On the other hand, adeno-associated virus vector serotype 9 carrying LECT2 short hairpin RNA (AAV9-LECT2-shRNA) can target mouse LECT2 to inhibit LECT2/Tie1 signaling, thereby inducing portal angiogenesis, suppressing hepatic sinusoidal capillarization, and alleviating liver fibrosis (90). Yuan Lin and Meng Xu further explored the effect of AAV9-LECT2-shRNA combined with rVEGF or bevacizumab in the targeted therapy of liver fibrosis in mice. The shortcomings of bevacizumab and rVEGF in regulating different microvessels in the treatment of liver fibrosis are made up for by AAV9-LECT2 shRNA, the combination of varying angiogenesis modulators further improves the therapeutic effect on liver fibrosis, and the side effects of bevacizumab combination therapy are relatively less (58). In comparison, vascular endothelial cell regulators are aimed at the changes of angiogenesis in the development of liver fibrosis, directly anti-fibrosis, and in combination with other drugs targeting metabolic disorders, inflammation, and other mechanisms; theoretically speaking, the complementary advantages are apparent, and it is a direction worth exploring.

## Conclusion and perspectives

As MAFLD has become the primary cause of liver fibrosis and one of the most common indications for LT worldwide, the global health problems caused by the MAFLD pandemic cannot be ignored. In the face of a considerable disease burden, lifestyle interventions have failed to control the prevalence of MAFLD effectively, and bariatric metabolic surgery is unsuitable as a first-line treatment. It is important to explore safe and effective drug treatment options. The occurrence of MAFLD and its liver fibrosis progression involves many complex factors and mechanisms, such as metabolic disorders, inflammation, immunity, and ECM formation. Some new drugs with multiple mechanisms of action have been discovered, such as FXR agonists that can regulate bile acid metabolism and inflammatory response and PPAR agonists that target metabolic disorders and inflammation simultaneously, but it is still challenging to achieve satisfactory results when these drugs are used alone. Hence, a strategy for combining different types of drugs is necessary. In recent years, appropriate drug combination therapy has mainly focused on driving factors such as metabolic disorders, inflammation, and oxidative stress. In the future, it is believed that there will be more explorations of multi-drug combination therapy strategies targeting different pro-fibrosis pathways and fibrosis regression mechanisms.

## Author contributions

QD and HB wrote the original draft and further revised it. JW and WS organized and created tables. XZo and JS drew the figure. YX, YC, and XZh were responsible for project administration, revising, and approving the manuscript. All authors contributed to the manuscript and approved the submitted version.
